# Low Temperature (15 °C) Reduces Bacterial Diversity and Prolongs the Preservation Time of *Volvariella volvacea*

**DOI:** 10.3390/microorganisms7100475

**Published:** 2019-10-20

**Authors:** Xiuling Wang, Shunjie Liu, Mingjie Chen, Changxia Yu, Yan Zhao, Huanling Yang, Lei Zha, Zhengpeng Li

**Affiliations:** 1 Institute of Edible Fungi, Shanghai Academy of Agricultural Sciences, Shanghai 201403, China; wangxiuling0828chris@gmail.com (X.W.); Jasonliu86@foxmail.com (S.L.); mjchen@saas.sh.cn (M.C.); ycx41529@163.com (C.Y.); yanghuanling@saas.sh.cn (H.Y.); zhalei@saas.sh.cn (L.Z.); lizp_ln@126.com (Z.L.); 2 College of Life Sciences, Shihezi University, Shihezi 832003, China

**Keywords:** *Volvariella volvacea*, food preservation, bacterial community, *Firmicutes*, *Solibacillus*

## Abstract

Straw mushroom (*Volvariella volvacea*) is the most commonly cultivated edible fungus in the world, but the challenges associated with the preservation have limited its marketability. Microbiology, especially bacteria, play a key role in the deterioration of food, this study aimed to reveal the succession of the bacterial community on the surfaces of *V. volvacea* fruit bodies under different temperature conditions. We amplified 16S rRNA genes of V4 regions, obtained the bacterial species information by using high-throughput sequencing technology, and analyzed the effects of environmental temperature and preservation time on bacterial communities. The relative abundances of *Firmicutes*, *Bacilli*, and *Bacillales* increased significantly when straw mushrooms began to rot. Furthermore, the relative abundances of *Paenibacillus*, *Lysinibacillus* and *Solibacillus,* which belong to *Bacillales*, increased with the decay of straw mushroom. The Shannon and Simpson indices of *V. volvacea* stored at 30 °C were significantly higher than those of *V. volvacea* stored at 15 °C, which indicates that a high temperature contributes to the improvement in the species diversity. According to the linear discriminant analysis (LDA) effect size (LEfSe) results, the number of biomarkers in the 30 °C group (32, 42.11%) was significantly higher than that in the 15 °C group (17, 22.37%), indicating that a high temperature has a clustering effect on some bacterial communities. A Spearman correlation analysis showed that *Pseudomonas*, *Stenotrophomonas* and *Solibacillus* promoted the decay of straw mushroom. In conclusion, a high temperature increases the bacterial diversity on the straw mushroom surfaces and has a clustering effect on the bacterial communities. The bacterial community consisting of *Firmicutes*, *Bacilli*, *Bacillales*, *Paenibacillus*, *Lysinibacillus*, *Pseudomonas*, *Stenotrophomonas* and *Solibacillus* could promote the decay of straw mushroom, so new preservation materials research can focus on inhibiting anaerobic and decay-causing bacteria to prolong preservation time.

## 1. Introduction

Straw mushroom (*Volvariella volvacea*), also known as Guangdong mushroom and Chinese mushroom, is one of the largest cultivated edible fungi in the world and one of the main exported mushrooms in China [1,2]. China began cultivating straw mushroom 300 years ago and was the first country to do so [3].

Straw mushroom is a kind of high-temperature mushroom that is not only delicious but also has a high nutritional value [4,5]. Its antioxidant, low-fat and high-protein properties make straw mushroom a typically healthy food in the public mind [6,7]. *V. volvacea* grows in hot and rainy environments in tropical and subtropical areas, and the optimum growth temperature of this mushroom is 30–32 °C, which is similar to the average summer temperature of its main production area. However, the high temperature is not conducive to the storage of the fruit body of *V. volvacea*. During transport, straw mushroom is placed in paper boxes at 5 kg per box. Due to the respiration of the picked *V. volvacea*, the temperature at the center of each package can be as high as 50 °C, which is extremely unfavorable for the preservation of *V. volvacea*. To date, there has been much advancement in *V. volvacea* cultivation, resulting in the ready availability of this mushroom [8,9,10,11], while the preservation of *V. volvacea* remains a serious problem. *V. volvacea* is unable to withstand low temperatures: when it is stored at 4 °C, the mycelium disintegrates and dies, and the fruit body softens and undergoes autolysis [12,13], which complicates the preservation of *V. volvacea*. At the same time, researchers found that the storage time of *V. volvacea* was significantly extended when the temperature was approximately 15 °C [3,14,15]. While it is expensive to maintain *V. volvacea* at the ideal storage temperature (15 °C), it is also difficult to control the temperature at the center of the box at 15 °C.

Numerous studies have shown that bacteria are an important reason for food spoilage and degradation [16,17,18,19,20]. To prolong the shelf life of the *V. volvacea* fruit bodies, this study focused on the bacterial community succession process on the surfaces of postharvest fruit bodies of *V. volvacea* and aimed to find a solution for *V. volvacea* fruit body preservation.

The bacterial community structures differ greatly among different stages of food spoilage, so investigation of the succession of the bacterial communities could help the identification of decay-causing bacteria [21]. To explore the succession of bacteria on the surfaces of *V. volvacea* fruit bodies during the process of spoilage, a high-throughput sequencing of the V4 16S rRNA gene was performed to identify bacterial species. The purpose of this research was to identify the bacterial community that played a key role in the process of *V. volvacea* fruit body decay, thus providing a reference for the preservation of *V. volvacea* fruit bodies.

## 2. Methods

### 2.1. Experimental Design and Simple Process

**Figure 1:**
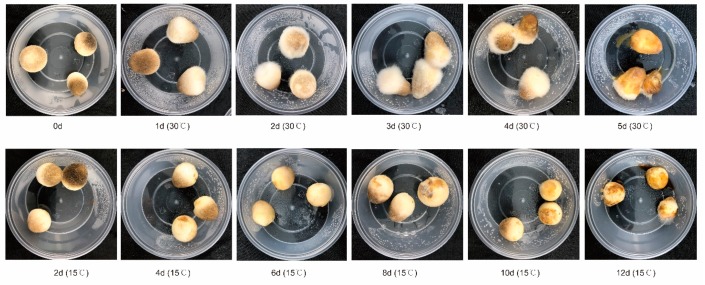
The sensory quality of the *V. volvacea* fruit bodies stored at 30 and 15 °C.

Considering the growth conditions of *V. volvacea*, we established 30 °C as the storage temperature of the fruitbody of *V. volvacea*. Additionally, previous studies have shown that 15 °C was an excellent temperature suitable for the storage of *V. volvacea*. Thus, this study used 15 and 30 °C as the storage temperatures for postharvest *V. volvacea*. We labeled the fresh fruit bodies of *V. volvacea* as the D0 group, and we used a letter-plus-number system (D0 group is not fully applicable because no letter was added in front of the group to indicate temperature) to label all the samples. The first letter in the label represents the storage temperature, i.e., A represents a storage temperature of 30 °C and B represents a storage temperature of 15 °C. The second letter, D, represents the day, the third character (the number between the letter and the decimal point) represents the storage time (number of days); and the fourth character represents the number of biological replicates. *V. volvacea* fruit bodies were stored at 30 °C for 5 days and sampled every 24 h, or stored at 15 °C for 12 days and sampled every 48 h (Figure 1). The mushrooms were sampled by selecting one sample that was close to the average state from the three *V. volvacea* fruit body samples in the crisper, i.e., the one that represented the overall preservation condition of all the *V. volvacea* fruit body samples was selected. The selected sample was then placed in a triangular flask containing 500 mL of double-distilled water and shaken for 3 min. The solution was filtered using filter paper with a 0.22-nm pore size, after which the filter paper was placed in a 50-mL centrifuge tube and stored at −80 °C.

### 2.2. Sequencing

#### 2.2.1. DNA Extraction

The total genomic DNA was extracted from microorganisms on the surfaces of the *V. volvacea* fruit body samples using the cetyl trimethyl ammonium bromide (CTAB) method. The DNA concentration and purity were quantified on 1% agarose gels (Life Technologies, Grand Island, NY, USA). Based on the determined concentration, the DNA was diluted to 1 ng/μL using sterile water.

#### 2.2.2. Amplicon Generation

The 16S rRNA genes from the distinct 16S V4 regions were amplified using the specific barcoded primers 515F-806R [22]. All PCRs were carried out with Phusion^®^ High-Fidelity PCR Master Mix (New England Biolabs, Ipswich, MA, USA).

#### 2.2.3. PCR Product Mixing and Purification

An equal volume of 1× loading buffer (containing SYBR Green) was mixed with the PCR products, and electrophoresis was performed on a 2% agarose gel for detection. The PCR products were mixed at equal concentrations. Then, the mixed PCR products were purified with the GeneJET™ Gel Extraction Kit (Thermo Scientific, Waltham, MA, USA).

#### 2.2.4. Library Preparation and Sequencing

Sequencing libraries were generated using an Ion Plus Fragment Library Kit with 48 reactions (Thermo Scientific, Waltham, MA, USA) following the manufacturer’s recommendations. The library quality was assessed on a Qubit 2.0 Fluorometer (Thermo Scientific, Waltham, MA, USA). Finally, the library was sequenced on an Ion S5TM XL platform, and 400 bp/600 bp single-end reads were generated.

### 2.3. Data Analysis

#### 2.3.1. Single-End Read Quality Control

The single-end reads were assigned to samples based on their unique barcode and truncated by removing the barcode and primer sequence. A quality filtering of the raw reads was performed using specific filtering conditions to obtain high-quality clean reads according to the cutadapt quality control process [23] (V1.9.1, http://cutadapt.readthedocs.io/en/stable/). The reads were compared to a reference database (SILVA database, https://www.arb-silva.de/) [24] using the UCHIME algorithm (http://www.drive5.com/usearch/manual/uchime_algo.html) [25] to detect chimera sequences, and the chimera sequences were then removed [26] to finally obtain the clean reads.

#### 2.3.2. OTU Cluster and Species Annotation

Sequence analysis was performed by UPARSE software (UPARSE v7.0.1001, http://drive5.com/uparse/) [27]. Sequences with ≥ 97% similarity were assigned to the same operational taxonomic unit (OTU). A representative sequence from each OTU was screened for further annotation. For each representative sequence, the SILVA database (Version 132) (https://www.arbsilva.de/) [24] was used, based on the Mothur algorithm, to annotate the taxonomic information. To study the phylogenetic relationship of different OTUs and to compare the dominant species among the different samples (groups), multiple sequence alignments were performed using the MUSCLE software (http://www.drive5.com/muscle/) [28]. The OTU abundance information was normalized using a standard sequence number corresponding to the sample with the fewest sequences. Subsequent analyses of the alpha diversity and beta diversity were all performed based on the normalized output data.

#### 2.3.3. Alpha Diversity

Alpha diversity is applied to analyze the complexity of the species diversity in a sample, using 6 indices: Observed species, Chao1, Shannon, Simpson, abundance-based coverage estimator (ACE), and Good’s coverage indices. For our samples, all these indices were calculated with QIIME (Version 1.7.0) and visualized with R software (Version 2.15.3).

Two indices were selected to identify the community richness: Chao with the Chao1 estimator (http://www.mothur.org/wiki/Chao) and ACE with the ACE estimator (http://www.mothur.org/wiki/Ace). Two indices were used to identify the community diversity: the Shannon index (http://www.mothur.org/wiki/Shannon) and the Simpson index (http://www.mothur.org/wiki/Simpson). Additionally, one index was used to characterize the sequencing depth: Good’s coverage (http://www.mothur.org/wiki/Coverage).

#### 2.3.4. Beta Diversity

A beta diversity analysis was used to evaluate the differences in the species complex among samples. The beta diversity of both the weighted and unweighted UniFrac distances were calculated by QIIME software (Version 1.7.0).

#### 2.3.5. Environmental Factor Correlation Analysis

In the Spearman correlation analysis, the Spearman correlation coefficient of the species and environmental factors was first calculated by the corr.test function of the psych package in R. Then, the significance of the results was evaluated and visualized by the pheatmap function in the heatmap package. The variance partitioning canonical correspondence analysis (VPA) is a type of partial analysis method. The rda function (X, Y, Z) in the vegan package was used to analyze the main environmental factor (Y) and the coenvironmental factor (Z) in the species distribution (X). The impact can quantify the effect of a certain type of environmental factor on the species distribution.

## 3. Results

### 3.1. Study of Bacterial Community Structure of Straw Mushroom in Response to Temperature and Preservation Time

**Figure 2:**
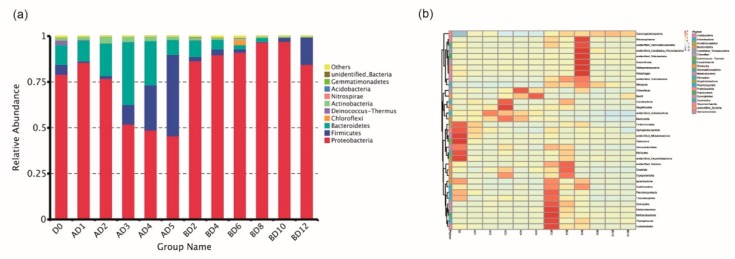
(**a**) Column chart showing the species relative abundances at the *Phylum*-level and (**b**) clustering heat map at the *Class*-level.

Based on the results of species annotation, we selected the top 10 species with the highest relative abundance at every classification level for each group and generated a column chart to visualize the relative abundances of the species. According to the relative abundance histogram at the *Phylum*-level, *Proteobacteria*, *Firmicutes* and *Bacteroidetes* occupied most of the bacterial community, and their abundances changed significantly at 30 °C (Figure 2a). In addition, the relative abundance of *Firmicutes* increased as the preservation time increased. The abundance information of each sample was clustered at the *Class*-level and plotted on a heat map using the R software pheatmap package, which can facilitate the discovery of the classes that were increased or decreased in the samples. The highest rank method was used to select the 35 most abundant classes. According to the results of the clustering heat map (Figure 2b), *Fimbriimonadia*, *Alphaproteobacteria*, *unidentified_Melainabacteria*, *Deinococcus*, *Verrucomicrobiae*, *Mollicutes*, and *unidentified_Oxyphotobacteria* accumulated on the surfaces of fresh straw mushrooms (D0). *Planctomycetacia*, *Thermoleophilia*, *Synergistia*, *Ktedonobacteria*, *Methanobacteria*, *Phycisphaerae*, and *Acidobacteriia* accumulated on the surfaces of the straw mushrooms in the BD2 group (15 °C, the second day). *Nitrososphaeria*, *unidentified_Gemmatimonadetes*, *unidentified*_*Candidatus*_*Woesebacteria*, *unidentified_Rokubacteria*, *Anaerolineae*, *Deltaproteobacteria* and *Holophagae* accumulated on the surfaces of straw mushrooms in the BD6 group (15 °C, the 6th day). Additionally, the relative abundance of *Gammaproteobacteria* on the surfaces of fresh straw mushrooms was very low.

**Figure 3:**
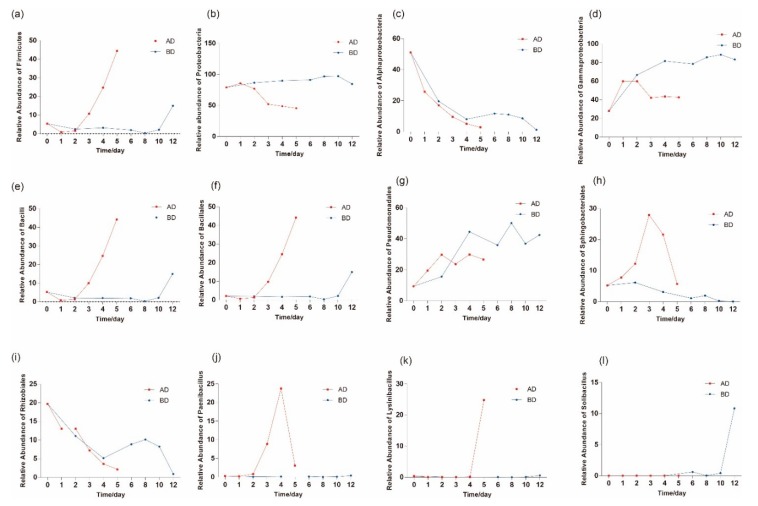
The bacterial species changed significantly with temperature and preservation time. (**a**) *Firmicutes*, (**b**) *Proteobacteria*, (**c**) *Alphaproteobacteria*, (**d**) *Gammaproteobacteria*, (**e**) *Bacilli*, (**f**) *Bacillales*, (**g**) *Pseudomonadales*, (**h**) *Sphingomonadales*, (**i**) *Rhizobiales*, (**j**) *Paenibacillus*, (**k**) *Lysinibacillus* and (**l**) *Solibacillus*. AD means 15 °C, BD means 30 °C.

The relative abundances of *Firmicutes* (Figure 3a), *Bacilli* (Figure 3e) and *Bacillales* (Figure 3f) increased significantly in the later period (at both 15 and 30 °C). *Paenibacillus* (Figure 3j), *Lysinibacillus* (Figure 3k) and *Solibacillus* (Figure 3l) belong to *Bacillales*; the relative abundances of these three genera also increased in the later period (15 or 30 °C).

The relative abundance of *Proteobacteria* was significantly reduced from the third day at only 30 °C (Figure 3b). The relative abundance of *Alphaproteobacteria* (Figure 3c) decreased with time, while that of *Gammaproteobacteria* (Figure 3d) increased with time. *Rhizobiales* (Figure 3i) and *Sphingomonadales* (Figure 3h) belong to *Alphaproteobacteria* (Figure 3c), and these three taxa exhibited similar changes overall. While the relative abundance of *Rhizobiales* (Figure 3i) changed significantly in the later period at only 30 °C, the relative abundance of *Sphingomonadales* (Figure 3h) changed significantly from the third to fifth days at only 15 °C. *Pseudomonadales* (Figure 3g) belongs to *Gammaproteobacteria* (Figure 3d), and the relative abundance of this taxon roughly increased with time.

### 3.2. Low Temperature (15°C) Reduces Bacterial Diversity

**Figure 4:**
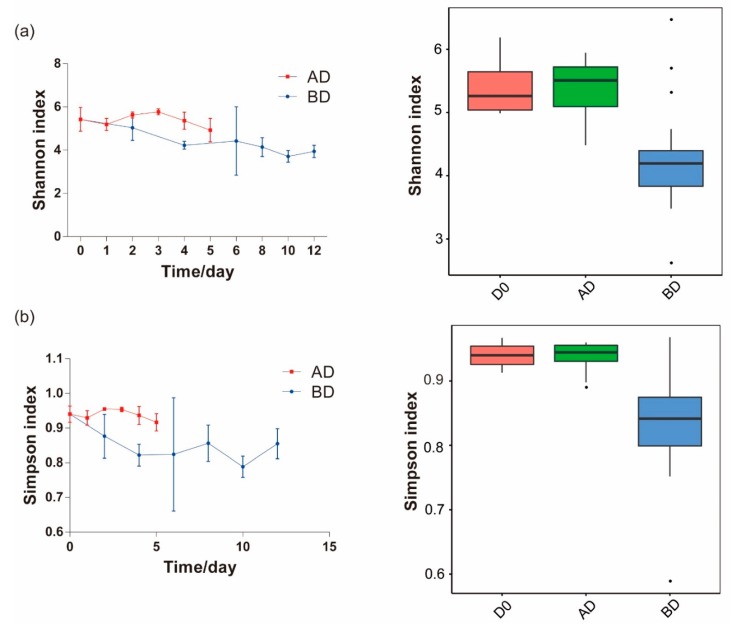
(**a**) The Shannon index and (**b**) Simpson index increase at high temperatures. D0 means 0-day, AD means 15 °C, and BD means 30 °C.

Taking the Shannon and Simpson indexes as an example (Table 1 and Table 2), these indexes were significantly higher for the bacteria from *V. volvacea* stored at 30 °C (AD) and fresh (D0) *V. volvacea* than for bacteria from *V. volvacea* stored at 15 °C (BD) (Figure 4). This finding indicates that low-temperature conditions reduce the diversity of the bacterial community. Furthermore, the bacterial diversity showed a downward trend over time at 15 °C (Figure 4). The alpha diversity indexes data for each group can be found in Table 1 and Table 2. Additionally, the good’s coverage index indicates that the sequencing depth was sufficient for this experiment.

As shown in the flower plots (Figure S1), the number of OTUs in AD2 and AD3 is greater than that in AD1, AD4, and AD5 (Figure S1b), while the values of BD8, BD10, and BD12 are significantly lower than those of BD2, BD4, and BD6 (Figure S1c). From the results of the sensory evaluation (Figure 1), at 30 °C, the food value of *V. volvacea* decreased from the second day. At 15 °C, the edible value of *V. volvacea* on the 8th day was significantly lower than that on the 6th day. When straw mushroom loses its edible value, its surface microbes exhibit intense succession, which is why some microbiological indicators are used for determining food safety.

**Figure 5:**
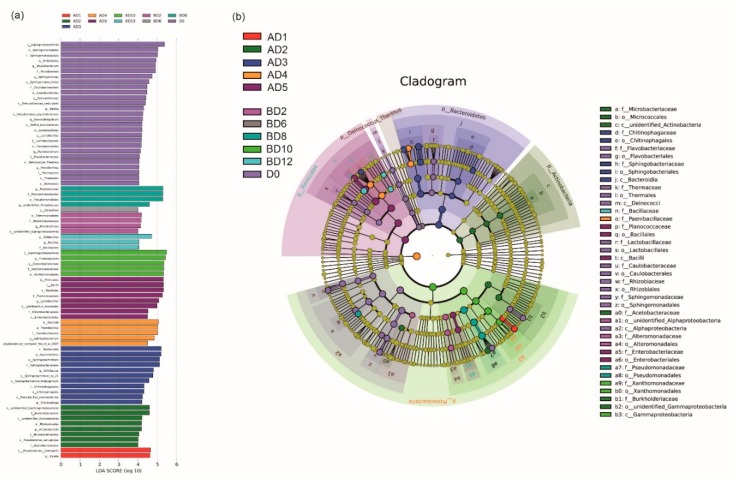
The (**a**) LDA (linear discriminant analysis) value distribution histogram and (**b**) evolutionary branch map. Description: The LDA value distribution histogram shows the species with an LDA scores greater than the set value (the default setting is 4), i.e., the biomarkers with statistical differences among the groups. Species with abundant significant differences among different groups are shown, and the length of the histogram represents the magnitude of the impact of different species (i.e., the LDA score). In the evolutionary branch diagram, the circle radiating from the inside to the outside represents the classification level from the *Phylum* to the *Genus* (or *Species*). Each small circle at a different classification level represents a classification at that level, and the diameter of the small circle is proportional to the relative abundance. Color scheme: species without significant differences are uniformly colored yellow; the differential species biomarkers are colored by group. The red nodes represent the microbial groups that play important roles in the red group, and the green nodes indicate microbial groups that play important roles in the green group. If a group is missing in the figure, there was no significant differences in that group. The species name represented by letters in the figure are shown in the legend on the right.

The linear discriminant analysis (LDA) effect size (LEfSe) results revealed statistically significant biomarkers among the groups, entailing species with significant differences among different groups. The LEfSe results contain three parts: the LDA value distribution histogram (Figure 5a), the evolutionary branch map (Figure 5b), and the biomarkers in different groups (Figure 5b). According to the LEfSe results, there were 27 biomarkers in the D0 group, accounting for 35.53% of the total biomarkers. Compared with the BD group (17, 22.37%), the AD group (32, 42.11%) had more species with significant differences. This result shows that the bacterial community changed greatly under high-temperature conditions, which means that the bacterial community was more sensitive to a high temperature (30 °C) than to low temperature (15 °C).

### 3.3. Environmental Factor Analysis

#### 3.3.1. Spearman Correlation Analysis

**Figure 6:**
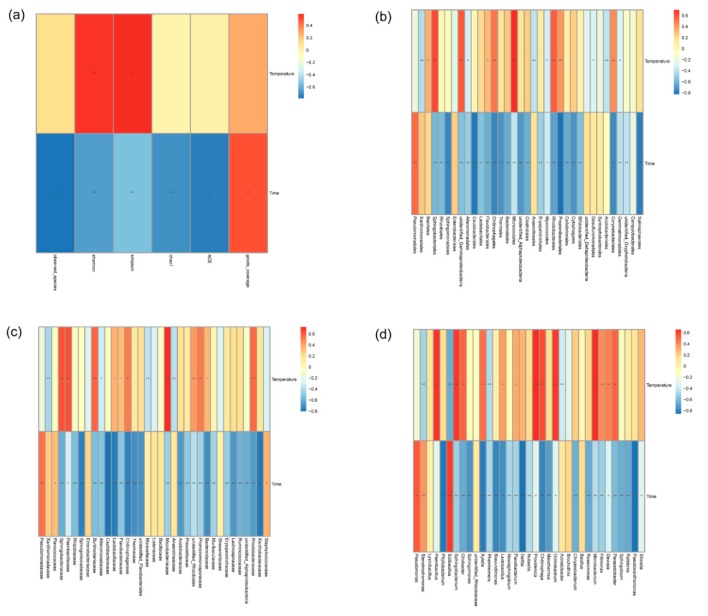
The heat maps of the Spearman correlation analysis. (**a**) Heat map of the Spearman correlation analysis of alpha diversity. (**b**) Heat map of the Spearman correlation analysis of bacterial abundance at the Order level. (**c**) Heat map of the Spearman correlation analysis of bacterial abundance at the Family level. (**d**) Heat map of the Spearman correlation analysis of bacterial abundance at the Genus level. Description: the vertical axis contains the environmental factor information, and horizontal axis is the species information. The intermediate heat map corresponds to the Spearman correlation coefficient r, with values between −1 and 1, with *r* < 0 indicating a negative correlation and *r* > 0 indicating a positive correlation; * indicates *p* < 0.05.

We used a Spearman rank correlation to explore the relationship between the environmental factors (time and temperature) and the alpha diversity indexes of the bacterial community. The heat map from the Spearman correlation analysis indicates that most of the diversity indicators were inversely proportional to time; that is, as the preservation time increased, the bacterial community diversity index decreased, indicating that the bacterial diversity on the surface of fresh straw mushroom was higher than that of decaying straw mushroom (Figure 6a). The Shannon index and Simpson index were proportional to the temperature, indicating that a higher temperature can lead to an increased bacterial diversity.

At the *Order* (Figure 6b), *Family* (Figure 6c) and *Genus* levels (Figure 6b), the relative abundance of most bacteria on the surface of *V. volvacea* was inversely proportional to the preservation time and proportional to the temperature. At the *Phylum* and *Class* levels, this phenomenon was not observed. The abundances of some bacterial genus (*Pseudomonas*, *Stenotrophomonas* and *Solibacillus*) were directly proportional to the preservation time, which indicates that as the straw mushrooms decayed, the abundances of these bacteria increased. It is believed that these bacterial communities may promote the decay of straw mushroom.

#### 3.3.2. Variance Partitioning Canonical Correspondence Analysis

**Figure 7:**
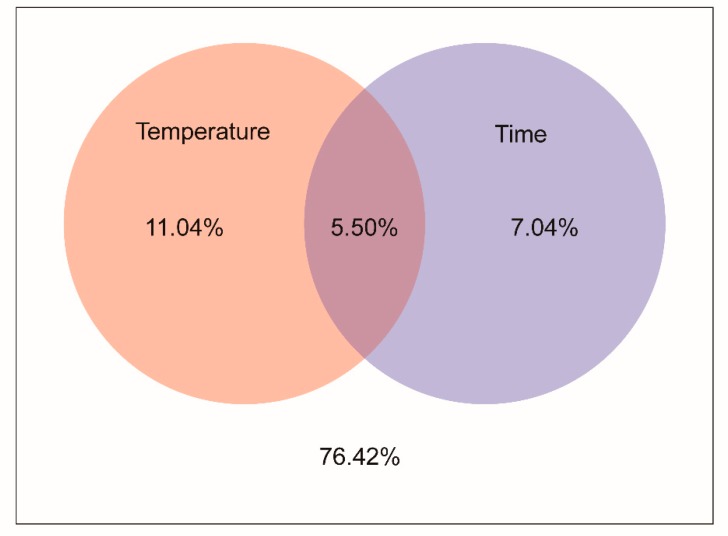
The VPA results. Description: the intersection of the circles is the explanatory quantity shared by the temperature factor and the time factor, and the area outside the circles is the unexplained quantity.

VPA focuses on interpreting the distribution of microbial communities by including various environmental factors. In this paper, we analyzed the OTU data of the bacterial community and obtain the interpretation of time and temperature on the community structure. The VPA results showed that temperature had a greater influence than time on the bacterial community structure of *V. volvacea* (Figure 7).

## 4. Discussion

The challenges associated with the preservation of *V. volvacea* limit its marketability. Temperature is an important environmental factor for food preservation and plays a key role in the preservation of *V. volvacea* [29]. Ideal preservation temperatures can greatly help extend the preservation time of food. Not only are bacteria directly related to the rooting of food, but bacterial communities are also among the important indicators used for evaluating the degree of food rot [30,31]. Studies on mushroom preservation have shown that new packaging materials can prolong the preservation time of food through inhibition of some specific bacteria [32,33]. The research results described in this paper revealed some bacterial communities associated with the decay of straw mushroom, such as *Pseudomonas*, *Stenotrophomonas*, *Lysinibacillus* and *Solibacillus,* providing research ideas and directions for the development of bacteriostatic packaging materials.

### 4.1. Preservation Time and Temperature Significantly Affected the Decay-Causing Bacteria

The relative abundance of *Firmicutes* increased significantly with prolonged preservation time of straw mushroom. *Bacillales* belong to *Bacilli* and *Bacilli* belong to *Firmicutes*; the relative abundance of these three changed consistently. *Paenibacillus*, *Lysinibacillus* and *Solibacillus* belong to *Bacillales*, and the relative abundance of *Paenibacillus* and *Lysinibacillus* increased at 30 °C, while the relative abundance of *Solibacillus* increased at 15 °C.

Dasgupta et al. [34] showed that *Paenibacillus lentimorbus* NRRLB-30488 belongs to *Paenibacillus,* and that *P. lentimorbus* NRRLB-30488*R* can produce both chitinase and β-1,3-glucanase enzymes in the culture medium; these ingredients promote the dissolution of polysaccharides such as lignin and cellulose [35]. Furthermore, the relative abundance of *Paenibacillus* in the AD3 and AD4 groups was very high. Based on the above analysis, we believe that *Paenibacillus* microorganisms may promote the decay of the fruit bodies of straw mushrooms. *Lysinibacillus xylanilyticus* belongs to *Lysinibacillus,* and it can degrade xylan [36]. We speculate that *Lysinibacillus* could promote food rot. Additionally, *Solibacillus silvestris,* which belong to *Solibacillus,* can degrade *N*-acyl-homoserine lactone [37], so *Solibacillus* likely promote the decay of straw mushroom. The above research shows that *Firmicutes* and *Bacillales* are closely related to the decay of straw mushroom, and that *Solibacillus* functions at 15 °C, while *Paenibacillus* and *Lysinibacillus* function at 30 °C.

As the storage time increased, the Shannon and Simpson index showed a downward trend. A possible reason for this is that some bacterial species disappeared due to the fact that the harsh environment (such as hypoxia) leaving some adaptable bacteria as the dominant bacterial community. According to the Spearman correlation analysis, the higher the temperature is, the higher the diversity of bacterial communities and the higher the relative abundance of microorganisms, while the shorter the preservation time is, the higher the relative abundance of bacteria. Meanwhile, the abundances of *Pseudomonas*, *Stenotrophomonas* and *Solibacillus* were directly proportional to the preservation time, which means that these bacteria may promote the decay of straw mushrooms. Previous literature also mentioned that *Pseudomonas* [38], *Stenotrophomonas* [39,40] and *Solibacillus* [37] are closely related to biodegradation and pathogen infection.

### 4.2. High Temperature Promotes the Reproduction of Microorganisms

Temperature affects the composition of bacterial communities on the surface of straw mushrooms [41]. At 15 °C, the alpha diversity index of the bacterial community of *V. volvacea* was initially lower than that at 30 °C, indicating that the bacterial diversity was low under low-temperature conditions. This finding indicates that a considerable portion of the bacterial community was inhibited when the storage temperature was 15 °C. This result suggests that the species diversity and richness of the bacterial community were higher under 30 °C than at 15 °C, indicating that high temperature conditions accelerate the decay of *V. volvacea*. Consequently, a low temperature (15 °C) had a significant preservation effect by reducing the bacterial diversity. Furthermore, the LEfSe results showed that the bacterial community on the surfaces of the *V. volvacea* fruit bodies had a large number of biomarkers (species with significant differences) at 30 °C, which shows that a high temperature has a clustering effect on certain bacterial communities.

The correlation analysis of the environmental factors showed that all of the alpha diversity indices were closely related to time, and the VPA results showed that the temperature had a greater influence on the bacterial community structure of the postharvest fruit bodies of *V. volvacea* than time did. The reason for the difference in the results is that VPA focuses on explaining the community distribution with environmental factors, while the Spearman rank correlation focuses on the relationship between environmental factors and alpha diversity.

To date, few studies have examined bacterial succession during the decay process of edible fungi, and for preservation of truffles, 4 °C was found to be the most efficient temperature [42]. Some related studies have mostly focused on microorganisms in compost at different stages [43,44,45]. There is a lack of similar studies because cryopreservation is a suitable storage method for most mushrooms. However, conventional cryopreservation (4 °C) can’t be used for *V. volvacea* fruit body preservation; therefore, we must consider other solutions. In this paper, we studied the bacterial community succession and hypothesized that the preservation of the *V. volvacea* fruit bodies could be prolonged by inhibition of decay-causing bacteria.

According to the results of this study, the preservation temperature is a key factor in the decay of straw mushrooms, so controlling the temperature at approximately 15 °C can effectively extend their preservation time. Additionally, research on new preservation materials can focus on the inhibition of the growth of decay-causing bacteria, such as *Firmicutes*, *Bacilli*, *Bacillales*, *Paenibacillus*, *Lysinibacillus*, *Pseudomonas*, *Stenotrophomonas* and *Solibacillus*.

## Figures and Tables

**Table 1:** The alpha diversity index (all samples).

Group	Observed Species	Shannon	Simpson	Chao1	ACE	Good’s Coverage	PD Whole Tree
D0	611	5.424	0.94	711.262	714.618	0.997	46.46
AD1	461	5.189	0.929	547.522	544.016	0.998	30.138
AD2	420	5.629	0.955	498.262	486.018	0.998	25.157
AD3	484	5.773	0.954	551.587	546.374	0.998	28.6
AD4	409	5.361	0.936	474.942	463.02	0.998	23.895
AD5	365	4.925	0.916	422.119	420.295	0.998	20.822
BD2	654	5.035	0.876	805.452	805.074	0.996	50.533
BD4	471	4.226	0.822	570.405	596.579	0.998	36.328
BD6	431	4.423	0.824	522.711	519.201	0.998	32.856
BD8	309	4.141	0.856	470.589	410.364	0.998	20.49
BD10	261	3.713	0.788	339.822	342.007	0.998	15.637
BD12	244	3.94	0.855	322.445	318.029	0.999	16.338

**Table 2:** The alpha diversity index (all groups).

Group	Observed Species	Shannon	Simpson	Chao1	ACE	Good’s Coverage	PD Whole Tree
AD	428	5.375	0.938	498.886	491.945	0.998	25.722
BD	395	4.246	0.837	505.238	498.542	0.998	28.697
D0	611	5.424	0.94	711.262	714.618	0.997	46.46

## Supplementary Materials

The following are available online at https://www.mdpi.com/2076-2607/7/10/475/s1.
