# Supplementary material for: Low Temperature (15 °C) Reduces Bacterial Diversity and Prolongs the Preservation Time of Volvariella volvacea
Source: Microorganisms. 2019 Oct 20;7(10):475. doi: 10.3390/microorganisms7100475 (PMC6843861; doi:10.3390/microorganisms7100475)
Supplement: Supplementary file 1 [file microorganisms-07-00475-s001.zip › microorganisms-595173-SI.docx]

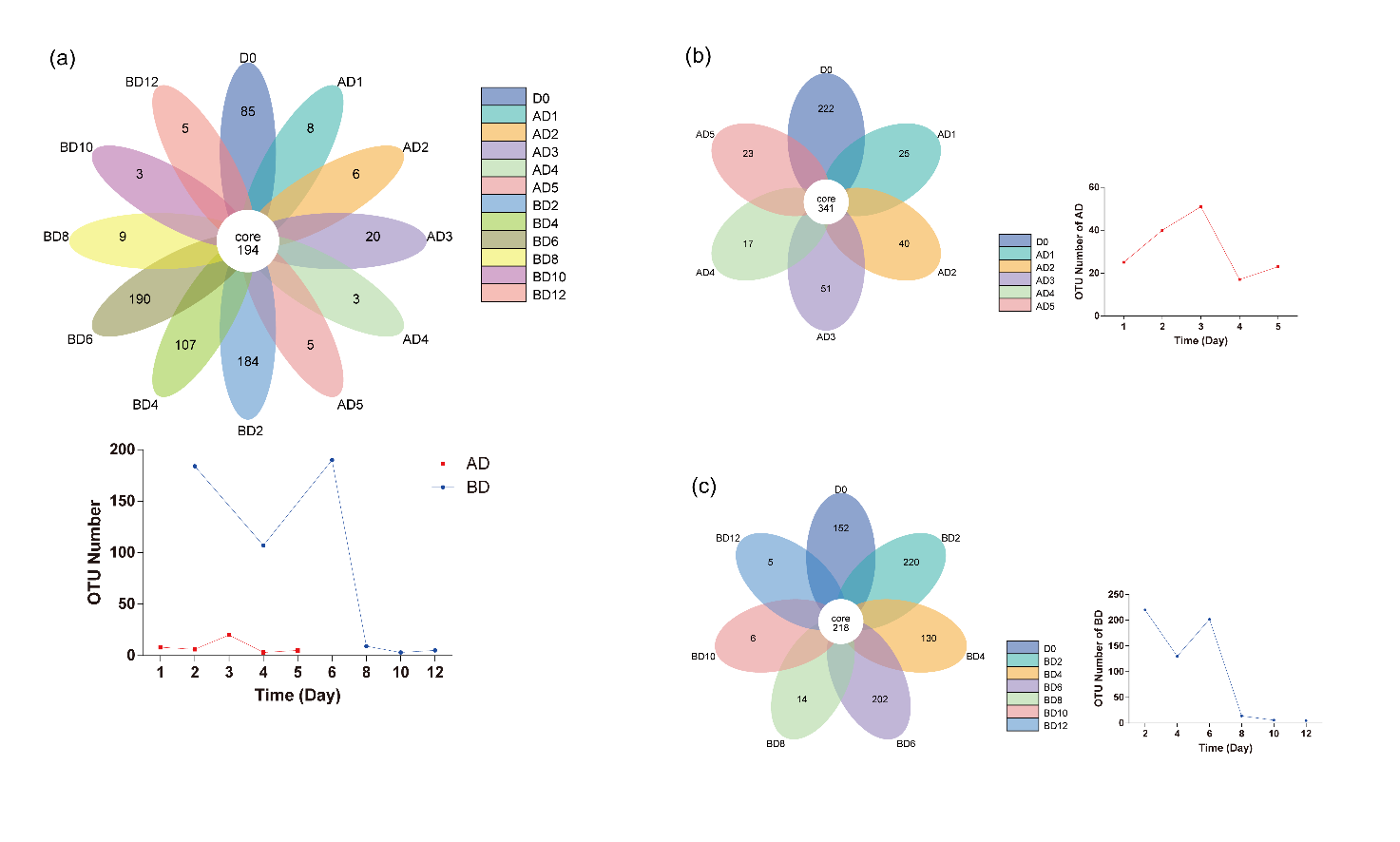


**Figure S1.** Flower plots under different groupings. Description: Each petal in the flower plots represents a group. Different colors represent different groups. The middle core number represents the total number of OTUs from all the groups. The numbers on the petals represent the number of unique OTUs of that group. 3.3. The bacterial community is more significantly different between groups under 30 °C conditions.
